# Metabolic and Lipidomic Assessment of Kidney Cells Exposed to Nephrotoxic Vancomycin Dosages

**DOI:** 10.3390/ijms221810111

**Published:** 2021-09-18

**Authors:** Simon Lagies, Roman Pichler, Georg Vladimirov, Jana Gawron, Fabian Bäzner, Annabell Schreiner, Dajana Kadena, Dietmar A. Plattner, Soeren S. Lienkamp, Bernd Kammerer

**Affiliations:** 1 Centre for Integrative Signalling Analysis, University of Freiburg, 79104 Freiburg, Germany; simon.lagies@zbsa.uni-freiburg.de (S.L.); georgvladimirov@web.de (G.V.); jana@gawron24.de (J.G.); fabian.baezner@gmx.de (F.B.); annabell-schreiner@gmx.de (A.S.); kadenadajana@yahoo.com (D.K.); 2 Institute of Organic Chemistry, University of Freiburg, 79104 Freiburg, Germany; dplatt@chemie.uni-freiburg.de; 3 Department of Medicine IV, Nephrology and Primary Care, Medical Center–University of Freiburg, Faculty of Medicine, University of Freiburg, 79110 Freiburg, Germany; roman.pichler@uniklinik-freiburg.de (R.P.); soeren.lienkamp@anatomy.uzh.ch (S.S.L.); 4 Berta-Ottenstein-Programme for Clinician Scientists, Faculty of Medicine, University of Freiburg, 79110 Freiburg, Germany; 5 Institute of Anatomy, University of Zurich, 8057 Zurich, Switzerland; 6 BIOSS Centre for Biological Signalling Studies, University of Freiburg, 79104 Freiburg, Germany

**Keywords:** vancomycin, nephrotoxicity, tubule, metabolomics, lipidomics, GC/MS, LC/MS, mass spectrometry

## Abstract

Vancomycin is a glycopeptide antibiotic used against multi-drug resistant gram-positive bacteria such as *Staphylococcus aureus* (MRSA). Although invaluable against resistant bacteria, vancomycin harbors adverse drug reactions including cytopenia, ototoxicity, as well as nephrotoxicity. Since nephrotoxicity is a rarely occurring side effect, its mechanism is incompletely understood. Only recently, the actual clinically relevant concentration the in kidneys of patients receiving vancomycin was investigated and were found to exceed plasma concentrations by far. We applied these clinically relevant vancomycin concentrations to murine and canine renal epithelial cell lines and assessed metabolic and lipidomic alterations by untargeted and targeted gas chromatography-mass spectrometry and liquid chromatography-mass spectrometry analyses. Despite marked differences in the lipidome, both cell lines increased anabolic glucose reactions, resulting in higher sorbitol and lactate levels. To the best of our knowledge, this is the first endometabolic profiling of kidney cells exposed to clinically relevant vancomycin concentrations. The presented study will provide a valuable dataset to nephrotoxicity researchers and might add to unveiling the nephrotoxic mechanism of vancomycin.

## 1. Introduction

Vancomycin is a glycopeptide antibiotic with activity against gram-positive bacteria, such as *Staphylococcus aureus* [1]. Since the emergence of methicillin-resistant *Staphylococcus aureus*, vancomycin is increasingly used in clinics with prolonged duration and elevated dosages. However, vancomycin harbors the risk of several adverse effects, which include ototoxicity [2], thrombocytopenia [3], and neutropenia [4]. Additionally, higher dosages of vancomycin are associated with an increased risk of acute kidney injury [5]. Hence, Du et al. used physiologically-based pharmacokinetic modeling and simulation and verified their results with a human specimen [6]. This study found vancomycin levels in the kidney which were up to 50-fold higher than plasma vancomycin levels. Therefore, vancomycin associated acute kidney injury is highly clinically relevant and the mechanisms behind it have only partially been unraveled. Wang and colleagues found that vancomycin activates microRNA-301a-5p in a methyl-CpG-binding domain protein 2 dependent manner, ultimately driving proximal tubule cells into apoptosis [7]. Similarly, Chen et al. uncovered stimulation of p53-dependent apoptosis by microRNA-192-5p in a human renal epithelial cell line upon vancomycin treatment [8]. Another mechanism to activate apoptosis in kidney cells used by vancomycin is the suppression of complex I, which leads to elevated levels of mitochondrial superoxide [9]. The production of reactive oxygen species (ROS) enhances the permeabilization of the mitochondrial membrane, resulting in apoptosome activation [10,11]. ROS also contribute to apoptosis by the formation of cardiolipin peroxides, a mitochondria specific phospholipid [12]. Hence, oxidative species also mediate expression of pro-inflammatory cytokines, as treatment with antioxidant species ameliorate vancomycin-induced nephrotoxicity [13].

A common consequence of mitochondrial redox imbalance is the activation of other redox dependent pathways. Specifically, the pharmacological inhibition of complex I results in an increase of glucose metabolization to lactic acid [14]. Complex I oxidizes reduced nicotinamide adenosine dinucleotide (NADH) and thus the inhibition of it causes increased levels of NADH [15]. Accumulated NADH is then oxidized by lactate dehydrogenase and replenishes the redox pool necessary to drive glycolysis [16]. Another redox dependent glucose metabolizing pathway is the polyol pathway, in which glucose is reduced to sorbitol, which is then oxidized to fructose [17]. The latter reaction also leads to the production of NADH [18]. Therefore, when the redox pool is shifted to elevated NADH, sorbitol cannot be oxidized to fructose anymore, resulting in an accumulation of sorbitol.

Despite the apparent role of mitochondria in vancomycin-induced acute kidney injury, no metabolomics studies of vancomycin treated kidney cells have been conducted so far. Du et al. used kidney cells’ supernatant after a 2,3-bis-(2-methoxy-4-nitro-5-sulfophenyl)-2H-tetrazolium-5-carboxanilide assay (XTT-assay) for an exometabolic screen based on reversed-phase chromatography mass spectrometry. They aimed at discovering potential bio-markers for vancomycin-induced nephrotoxicity and found several lyso-phospholipids in the supernatant of cells with low viability [6].

In regard with other nephrotoxins, metabolomics is widely used. The well-known chemotherapeutic drug cis-diamminedichloroplatinum II (cisplatin) is nephrotoxic [19] and has a high impact on renal metabolism. Among others, we showed accumulation of glucose and down-regulation of glycolysis intermediates together with a loss of amino acids [20]. Cyclosporine A is an immunosuppressive drug with adverse activity against the kidney [21]. Metabolic profiling of cultured kidney cells treated with different doses of cyclosporine A revealed a stark induction of glutathione metabolism, as well as alterations in amino acids and tricarboxylic acid cycle (TCA-cycle) intermediates [22]. Hence, the radiocontrast agent diatrizoic acid was also shown to induce an oxidative stress response [23]. Administration of the antiviral drug acyclovir, commonly causing acute kidney injury to rats, revealed excessive excretion of nitrogen containing metabolites to the urine [24]. Metabolomics is also capable of detecting alterations induced by non-toxic pathological stress conditions, such as high glucose and protein levels present in diabetic kidney disease [25].

In the presented study, we analyzed for the first time cellular changes in the metabolome and lipidome of kidney cell lines exposed to clinically relevant doses of vancomycin. We uncovered increased glycolysis and sorbitol levels, which might stem from the known mitochondrial disturbances caused by vancomycin. 

## 2. Results and Discussion

### 2.1. Vancomycin Is Taken Up in a Dose-Dependent Manner

**Figure 1:**
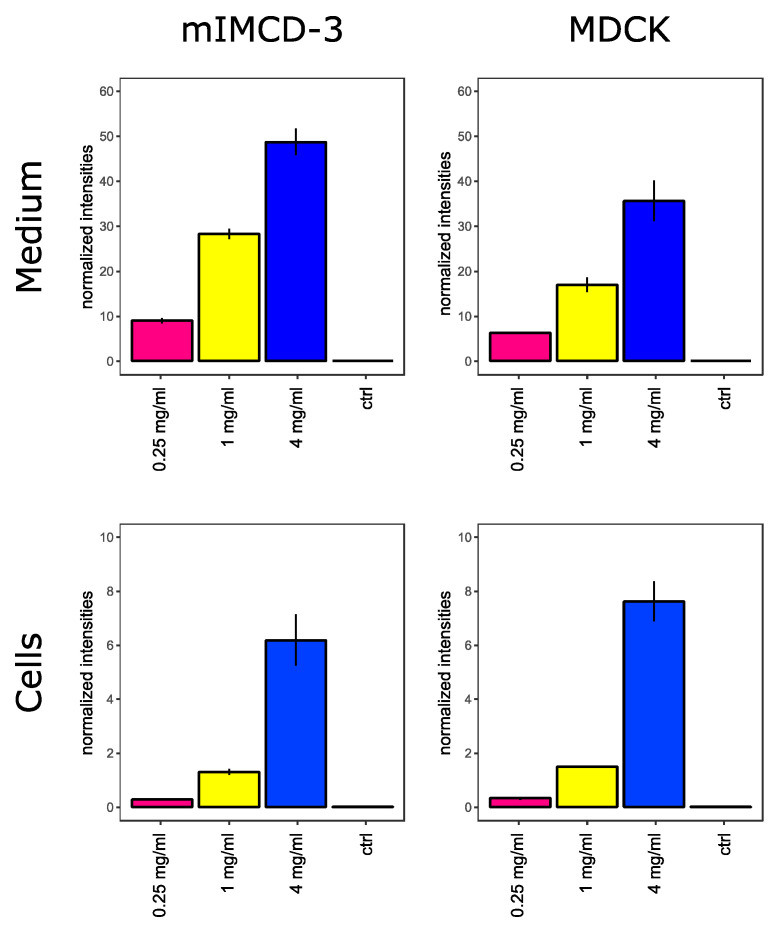
Vancomycin in cells and cell culture medium. Extracellular (**top**) and intracellular (**bottom**) vancomycin levels in mIMCD-3 cells (**left**) and MDCK cells (**right**) are shown. Y-axes show intensities which were acquired by liquid chromatography-mass spectrometry (LC/MS) and normalized to phenol red or O-methyl-L-tyrosine in cell culture media or within the cells, respectively. Error bars indicate standard deviation. N = 3.

To assess metabolic alterations induced by highly concentrated vancomycin, we treated mouse inner medullary collecting duct cells (mIMCD-3 cells) and Madin–Darby canine kidney cells (MDCK cells) with control media and media containing 0.25 mg/mL, 1 mg/mL or 4 mg/mL vancomycin. During these experiments, no major changes regarding morphology or cell number were observed (data not shown). As displayed in Figure 1, both cell lines have taken up vancomycin in a dose-dependent manner.

### 2.2. Metabolic Profiling of Vancomycin Treated Kidney Cell Lines

**Figure 2:**
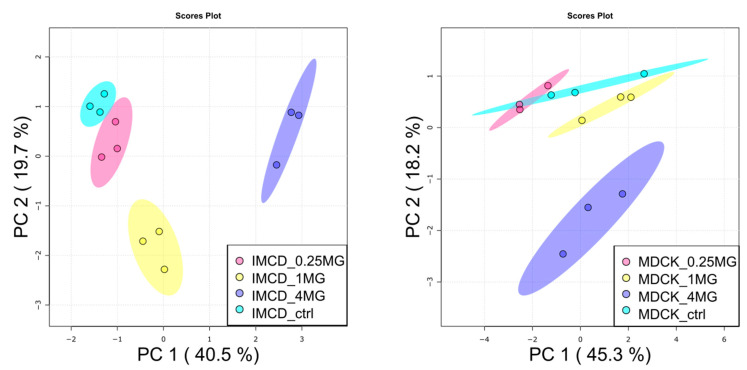
Principal component analysis of metabolites in vancomycin treated kidney cells. (**Left**): mIMCD-3 cells showed a dose-dependent impact on the metabolome by vancomycin. (**Right**): in MDCK cells, high-dose vancomycin was necessary to induce global alterations. PC: principal component. Turquois: control, red: 0.25 mg/mL vancomycin, yellow: 1 mg/mL vancomycin, blue: 4 mg/mL vancomycin. Dots represent samples, shaded area: the confidence interval. N = 3.

After proving the uptake of vancomycin by the used kidney cell lines, we subjected metabolite extracts to untargeted metabolic profiling by gas chromatography-mass spectrometry (GC/MS). Additionally, glutathione levels and small chain acyl-carnitines were acquired by targeted LC/MS analysis. In total, 88 metabolites were thoroughly identified using both mass spectra and retention time/index information. The data set was first analyzed by principal component analysis (PCA) as shown in Figure 2. Treatment with 0.25 mg/mL vancomycin did not result in global alterations, neither in mIMCD-3 cells nor in MDCK cells. Such alterations were visible with 1 mg/mL only in mIMCD-3 cells, and in both cell lines, when a concentration of 4 mg/mL was applied.

**Figure 3:**
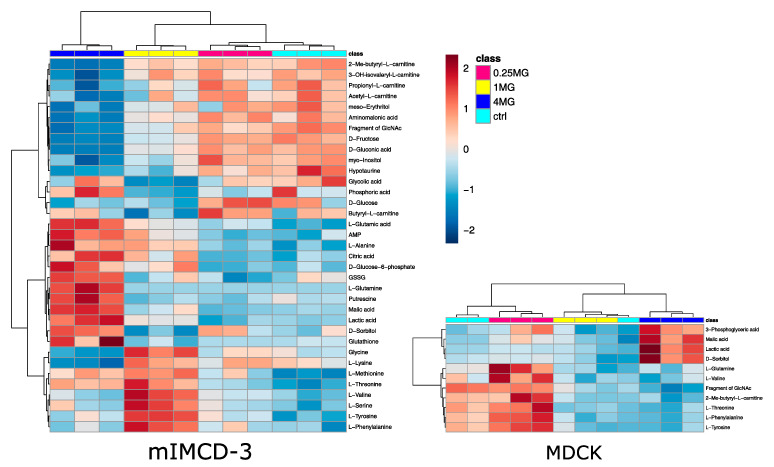
Heat map analysis of endometabolites. Only significantly altered metabolites (one-way ANOVA, corrected for multiple testing by FDR, *q*-value < 0.05) are displayed in mIMCD-3 cells (**left**) and MDCK cells (**right**). Cluster analysis after Euclidian and Ward are displayed on top for samples and on the left side for metabolites. Turquois: control, red: 0.25 mg/mL vancomycin, yellow: 1 mg/mL vancomycin, blue: 4 mg/mL vancomycin. Range scaled z-scores are displayed. N = 3.

Statistical analysis revealed 35 significantly altered metabolites (one-way analysis of variance (ANOVA), corrected for multiple testing by false discovery rate (FDR), *q*-value < 0.05) in mIMCD-3 cells and 11 significantly altered metabolites in MDCK cells. Detailed results of statistical analyses can be found in supplementary Table S1. These numbers also reflect the more profound impact of vancomycin on mIMCD-3 cells in comparison to MDCK cells as seen in the PCA (Figure 2). In Figure 3, these significant alterations are displayed in heat maps, in which range-scaled z-scores are shown. In both cell lines, distinct clustering is visible between control and 4 mg/mL vancomycin treatment.

In mIMCD-3 cells, small-chain acyl carnitines were down-regulated upon high-dose vancomycin treatment together with sugars and some sugar alcohols. Upregulated metabolites included key metabolites of glycolysis and the tricarboxylic acid cycle. In contrast to myo-inositol and meso-erythritol, the sugar alcohol sorbitol was up-regulated upon vancomycin exposure. A cluster of amino acids, which were mainly essential amino acids, trended to increase in 4 mg/mL vancomycin compared to the control, but they peaked in the 1 mg/mL vancomycin condition. An up-regulation of glycolysis and lactic acid in particular can point to a decreased activity of oxidative phosphorylation [26]. A disturbed activity in oxidative phosphorylation was already described and substantiates our finding [9]. A decreased TCA-cycle flux might also explain accumulating citrate levels. The decrease in acyl-carnitines further supports this hypothesis, since fatty acid oxidation is a predominant energy source in tubule cells [27]. An elevated usage of glucose also fits with the accumulation of sorbitol in tubule cells [25]. In line with that, we detected higher levels of sorbitol along with increased intermediates of glycolysis in 4 mg/mL vancomycin treated mIMCD-3 cells. In addition, sorbitol acts as an osmolyte in the kidney [28]. Thus, higher doses of vancomycin in the cell culture medium could also contribute to an increase of sorbitol. However, this is probably a minor contribution given the high osmolar pressure normally applied to renal epithelium [29]. Activation of sorbitol accumulation is corroborated by a dose-dependent decline in myo-inositol levels, a known effect of high sorbitol pathway activity [30]. The fact that fructose was decreased might suggest sorbitol accumulation was caused by redox imbalance [31]. Indeed, glutathione and glutathione disulfide increased in parallel, which points to an increased synthesis in response to redox imbalance (Figure 3 left). Elevated glutathione synthesis was already associated with another nephrotoxin, namely cyclosporine A [22].

In MDCK cells, some essential amino acids, tended to decrease with higher vancomycin doses. Four metabolites were significantly elevated in the 4 mg/mL vancomycin condition in comparison to the control cells. Three of them were catabolites of glucose, and lactic acid and sorbitol were likewise regulated as in the mIMCD-3 cell line. Together with the low levels of one small-chain acyl carnitine, these results confirm the finding from the mIMCD-3 cells: an impaired mitochondrial oxidative phosphorylation might have increased glycolysis, with parallel sorbitol accumulation. 

### 2.3. Lipidomics of Vancomycin Treated Kidney Cell Lines

**Figure 4:**
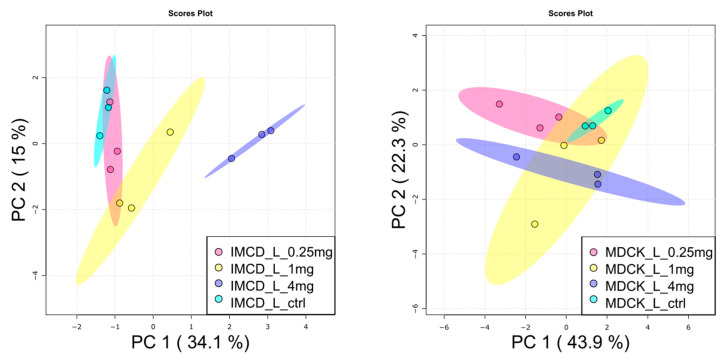
Principal component analysis of lipids in vancomycin treated kidney cells. (**Left**), mIMCD-3 showed dose-dependent alterations of lipids after vancomycin application. (**Right**): Lipids in MDCK cells were only impacted to a minor extent by vancomycin treatment. Turquois: control, red: 0.25 mg/mL vancomycin, yellow: 1 mg/mL vancomycin, blue: 4 mg/mL vancomycin. Dots represent samples, shaded area the confidence interval. N = 3.

Next, the lipophilic extracts were analyzed by targeted lipidomic analysis. Marked differences were unveiled in the baseline lipidomic profile of the two cell lines (Supplementary Figure S1). Additionally, the two cell lines behaved differently towards vancomycin exposure: only slight differences were observed in the PCA of MDCK cells between the control and 4 mg/mL vancomycin, whereas in mIMCD-3 cells, these conditions were clearly separated from each other (Figure 4).

**Figure 5:**
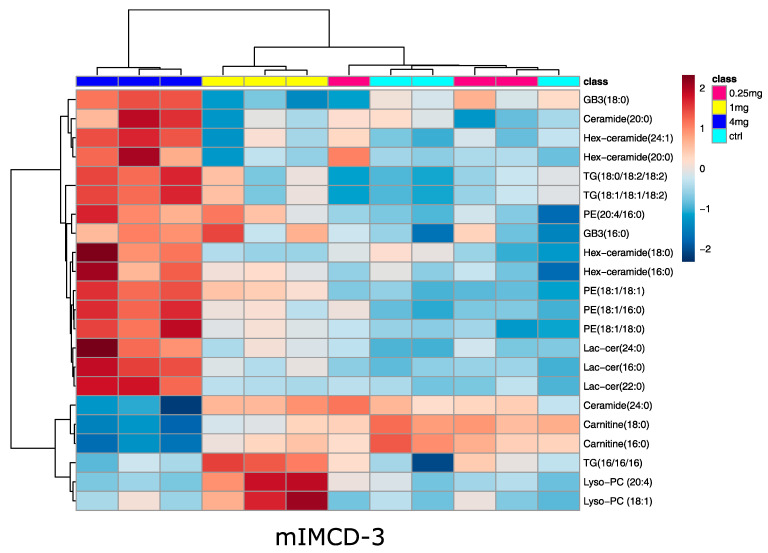
Heat map analysis of significantly altered lipids in mIMCD-3 cells. Significance was determined by ANOVA (one-way ANOVA, corrected for multiple testing by FDR, *q*-value < 0.05). Cluster analysis after Euclidian and Ward revealed marked differences in the high-dose vancomycin condition. Turquois: control, red: 0.25 mg/mL vancomycin, yellow: 1 mg/mL vancomycin, blue: 4 mg/mL vancomycin. Range scaled z-scores are displayed. N = 3.

In line with that, no significant alterations in lipid species upon vancomycin treatment were detected in MDCK cells, while 22 lipids were significantly altered in mIMCD-3 cells: in accordance with the metabolite analysis, two long chain acyl-carnitines were decreased in the 4 mg/mL condition (Figure 5). This confirms a reduced fatty acid oxidation in this cell line. Several lipids were up-regulated in mIMCD-3 cells, mainly glycosphingolipids and phosphatidylethanolamines (Figure 5). Activation of glycosphingolipid synthesis was already found in cisplatin induced acute kidney injury [32]. Four of the up-regulated glycosphingolipids are hexosyl-ceramides. Glucosyl-ceramides are known to be altered in several kidney diseases [33] and are involved together with other glycosphingolipids in apoptosis signaling [34,35]. Although we did not observe huge differences of cell mass, this might already prime the cells to enter into apoptosis. 

Phosphatidylethanolamines were increased upon high-dose vancomycin (Figure 5). Previous studies showed a decrease in phosphatidylethanolamines using aristolochic acid I, celiptium, and cisplatin [36,37,38], but in the latter case, cisplatin led to an increase of certain phosphatidylethanolamines in the renal medulla. This indicates that the regulation of phosphatidylethanolamines might be more complex in acute kidney injury and more toxins have to be tested to evaluate whether or not this lipid species is commonly regulated. Most of the lipids altered in mIMCD-3 cells upon vancomycin treatment were glycosphingolipids, which were increased. These lipids already had a higher basal level in the MDCK cell line, which might explain why these lipids did not further increase when vancomycin was applied. However, the lipidomic results from the mIMCD-3 cells should not be overinterpreted since they were not observed in MDCK cells.

## 3. Conclusions

To the best of our knowledge, this is the first metabolomics study of kidney cells treated with clinically relevant concentrations of the nephrotoxin vancomycin. These concentrations were only recently modelled by Du et al. and were found to exceed typical plasma concentrations up to 50-fold. We showed that these high concentrations were still dose-dependently taken up by the two kidney cell lines used. Further, although MDCK cells responded less than mIMCD-3 cells, both seemed to upregulate anaerobic glycolysis. This might be due to disturbances of the redox pool, which were already shown by Arimura et al. [9]. In line with an altered redox state and a consequent malfunctioning of mitochondria, is an increase in anaerobic glycolysis [26]. In addition, glucose metabolism towards fructose is disturbed in parallel to redox imbalance, which might explain increased sorbitol levels in both cell lines, and resulting from that, a decrease of myo-inositol [25]. The latter metabolite was however only altered in mIMCD-3 cells. A reduced functionality of mitochondria can also be assumed by the decrease in acyl-carnitines in high-dose vancomycin cells. Acyl-carnitines serve as shuttles for fatty acids, making them available for oxidation in mitochondria [39].

Lipid profiling revealed marked differences between the two cell lines and might have contributed to the decreased vulnerability of MDCK cells towards vancomycin on the lipidomic level. Therefore, the detected differences of glycosphingolipids should be critically evaluated, as they were only observed in one cell line. Future studies should investigate the metabolic effects of vancomycin in animal models to further understand the pathological mechanisms underlying vancomycin-induced acute kidney injury.

In conclusion, this study provides a new data set of several altered and unaltered metabolites and lipids in two established kidney cell lines treated with high concentrations of vancomycin. This might aid in developing new hypotheses of mechanisms of action in vancomycin induced nephrotoxicity.

## 4. Materials and Methods

### 4.1. Cell Culture

Mouse inner medullary collecting duct cells (mIMCD-3, ATCC^®^ CRL-2123™, ATCC, Manassas, VA, USA) were grown in Dulbecco’s modified Eagle’s medium (DMEM)/F12 medium (Gibco™) containing penicillin/streptomycin, and 10% fetal bovine serum (FBS). Madin-Darby canine kidney cells (MDCK, ATCC^®^ CCL-34™, ATCC, Manassas, VA, USA) were grown in DMEM (Gibco™) containing penicillin/streptomycin, and 10% FBS. Both cell lines were seeded in 6-well plates and grown until confluency. Cells were washed once with phosphate buffer saline before treatment with vancomycin. 

For treatment, 48 mg vancomycin hydrochloride (Hikma Pharmaceuticals, London, UK) were dissolved in 12 mL of the respective cell culture medium to yield a 4 mg/mL solution. This solution was sterile filtered and diluted with a corresponding cell culture medium to 1 mg/mL and 0.25 mg/mL. The cells were incubated with 2 mL of vancomycin containing (4, 1 and 0.25 mg/mL) or the control cell culture medium for 24 h. 

### 4.2. Cell Harvest

The cells were harvested as previously described [40]. In brief, cell culture medium was centrifuged to remove cell debris, transferred to a new vial and snap frozen in liquid nitrogen. Cells were washed twice with 0.9% NaCl and quenched with 1 mL ice-cold methanol:water (1:1, *v:v*) containing internal standards. Cells were scraped off, transferred to a new vial, and snap frozen. Cell culture medium and cells were stored at −80 °C until analysis.

### 4.3. Analysis of Vancomycin in Cell Culture Media

100 µL of cell culture medium were mixed with 900 µL ice-cold acetonitrile:methanol (3:1, *v:v*), vortexed and centrifuged (45 min, 20,000× *g*, 4 °C). 100 µL of the supernatant were evaporated in a speedvac and the pellets reconstituted in 100 µL ddH_2_O. 70 µL were transferred into an LC-Vial and 20 µL was used to prepare a mixed quality control sample. Vancomycin was analyzed by reversed-phase chromatography coupled to mass spectrometry using a gradient of water/0.1% formic acid with acetonitrile/0.1% formic acid (Waters: Acquity Hsst3 2.1 × 100 mm, 1.8 µm. Agilent Technologies, Waldbronn, Germany: G4220A, G4226A, G1316A, G6460A triple-quadrupole mass spectrometer). Gas temperature was set to 350 °C, gas flow was 8 L/min, and sheath gas temperature was 250 °C with 5 L/min flow. The nebulizer pressure was maintained at 30 psi. Capillary voltage was 3000 V in positive ionization mode with 500 V nozzle voltage. Samples were injected in a randomized order with regular injections of quality control samples to monitor possible analytical drifts.

### 4.4. Cell Lysis for Metabolomics and Lipidomics

500 µL chloroform (containing heptadecanoic acid as internal standard) was added to the methanol:water cell suspension and lysed by rigorous vortexing. Afterward, phases were separated by centrifugation. 300 µL of the upper phase were evaporated for GC/MS analysis, 300 µL of the upper phase were evaporated for LC/MS analysis and 200 µL of the lower phase were evaporated for lipidomics analysis.

### 4.5. Analysis of Glutathione and Small Chain Acyl-Carnitines

Glutathione and glutathione disulfide were analyzed as described by Schlimpert et al. [41] Authentic standards of small chain acyl-carnitines were used to determine retention times and multiple reaction monitoring (MRM) transitions and added to the existing method. MRM-transitions were determined with the MRM-optimizer software by Agilent Technologies.

### 4.6. Untargeted GC/MS Profiling

Untargeted metabolic profiling was conducted as previously described [20]. In brief, pellets were derivatized by methoxyamination and silylation and injected on an HP5-MS column. Gas chromatography was coupled with an electron ionization mass spectrometer. Data files were deconvoluted and peak picking performed by AMDIS [42]. Features were aligned with the online tool SpectConnect [43]. Metabolites were identified by mass spectra and retention indices from three different libraries [44,45,46] and an in-house data base.

### 4.7. Targeted Lipid Profiling by LC/QqQ-MS

Lipids were analyzed by targeted LC/MS MRM-analysis (Waters: BEH C18 2.1 × 100 mm, 1.8 µm. Agilent Technologies, Waldbronn, Germany: G4220A, G4226A, G1316A, G6460A triple-quadrupole mass spectrometer). Chromatographic separation was as previously described [40]. The gas temperature was set to 290 °C with a flow rate of 10 L/min. The sheath gas flow rate was 11 L/min at 370 °C. The nebulizer pressure was 25 psi. The mass spectrometer was operated with +5 kV/−4 kV and 500 V nozzle voltage. Details about transitions and collision energies are shown in Supplementary Table S2. The samples were kept at 15 °C and 5 µL were injected in randomized order with regular quality control samples in between.

### 4.8. Data Processing and Statistical Analysis

Intensities of metabolites or lipids were normalized to an internal standard and by the sum of all peaks [47]. MetaboAnalyst 5.0 were used for statistical analyses [48]. Missing values were replaced by one fifth of the minimal values of each feature. Samples with >25% relative standard deviation in the quality control samples were excluded from analysis. For principal component analysis and heat map generation, values were range-scaled. One-way ANOVA was used to determine significance, followed by false discovery rate-based multiple testing correction. A *q*-value cut-off of 0.05 was used. ANOVA was followed by Tukey’s post-hoc test. Results of statistical analyses are show in Supplementary Table S1.

## Data Availability

Metabolomics and Lipidomics results are provided in supplementary Tables S3 and S4, respectively.

## Supplementary Materials

The following are available online at https://www.mdpi.com/article/10.3390/ijms221810111/s1, Figure S1: heat map of all detected lipids, Table S1: Results of statistical analyses, Table S2: MRM transitions of lipids, Table S3: Results of metabolic analysis, Table S4: Results of lipidomic analysis.
