# Supplementary material for: Metabolic and Lipidomic Assessment of Kidney Cells Exposed to Nephrotoxic Vancomycin Dosages
Source: Int J Mol Sci. 2021 Sep 18;22(18):10111. doi: 10.3390/ijms221810111 (PMC8466248; doi:10.3390/ijms221810111)
Supplement: Supplementary file 1 [file ijms-22-10111-s001.zip › Supplementary information.pdf]

International Journal of *Molecular Sciences*

MDPI

Article

Metabolic and Lipidomic Assessment of Kidney Cells Exposed to Nephrotoxic Vancomycin Dosages – Supplementary information

Simon Lagies [1,2], Roman Pichler [3,4], Georg Vladimirov [1], Jana Gawron [1], Fabian Bäzner [1], Annabell Schreiner [1], Dajana Kadena [1], Dietmar A. Plattner [2], Soeren S. Lienkamp [3,5] and Bernd Kammerer [1,2,6,*]

1 Centre for Integrative Signalling Analysis, University of Freiburg, 79104 Freiburg, Germany; simon.lagies@zbsa.uni-freiburg.de (S.L.); georgvladimirov@web.de (G.V.); jana@gawron24.de (J.G.); fabian.baezner@gmx.de (F.B.); annabell-schreiner@gmx.de (A.S.); kadenadajana@yahoo.com (D.K.)

2 Institute of Organic Chemistry, University of Freiburg, 79104 Freiburg, Germany; dplatt@chemie.uni-freiburg.de

3 Department of Medicine IV: Nephrology and Primary Care, Medical Center – University of Freiburg, Faculty of Medicine, University of Freiburg, 79110 Freiburg, Germany; roman.pichler@uniklinik-freiburg.de (R.P.); soeren.lienkamp@anatomy.uzh.ch (S.S.L.)

4 Berta-Ottenstein-Programme for Clinician Scientists, Faculty of Medicine, University of Freiburg, 79110 Freiburg, Germany

5 Institute of Anatomy, University of Zurich, 8057 Zurich, Switzerland

6 BIOSS Centre for Biological Signalling Studies, University of Freiburg, 79104 Freiburg, Germany

* Correspondence: bernd.kammerer@zbsa.uni-freiburg.de

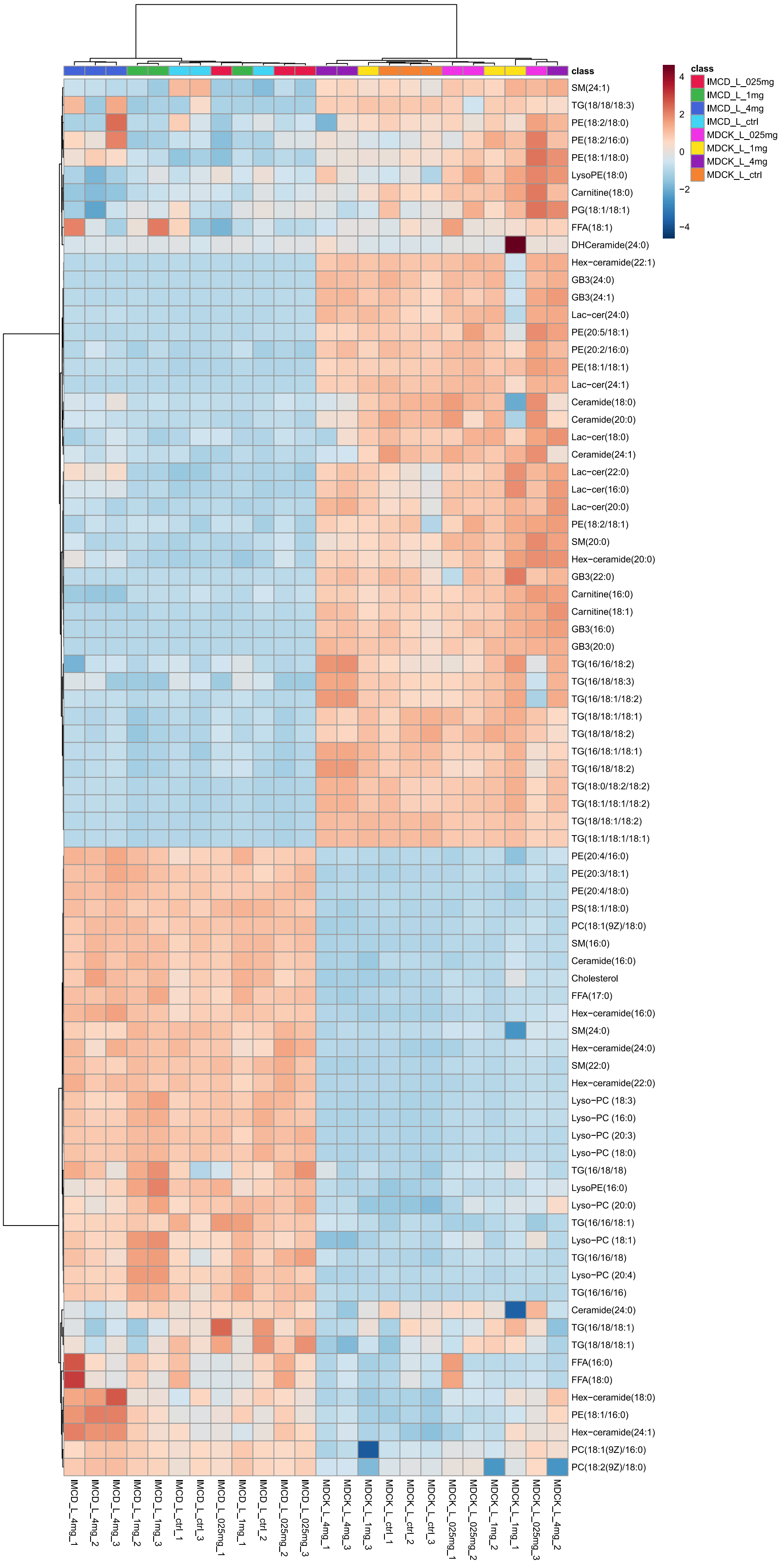

Figure S1: Heat map of all detected lipid species. Ranged scaled z-scores are displayed.
